# Administration of mycophenolic acid is not associated with malformations in descendants from kidney transplanted males

**DOI:** 10.1371/journal.pone.0202589

**Published:** 2018-09-12

**Authors:** Isabel Lopez-Lopez, Cristian Rodelo-Haad, Maria Luisa Agüera, Rosario Cabello-Jabalquinto, Elvira Esquivias-Motta, M. Dolores Navarro, Pedro Aljama, Alberto Rodriguez-Benot

**Affiliations:** 1 Maimonides Biomedical Research Institute of Cordoba (IMIBIC), Cordoba, Spain; 2 Reina Sofia University Hospital, Cordoba, Spain; 3 University of Cordoba, Department of Medicine, Cordoba, Spain; Icahn School of Medicine at Mount Sinai, UNITED STATES

## Abstract

**Background:**

In pregnant women, the use of Mycophenolic acid (MPA) is associated with teratogenicity. Recently, the European Medicines Agency (EMEA) and the Spanish Agency of Medicine and Sanitary Products (AEMPS) warned about the potential teratogenic effects of MPA. These adverse events may occur even in children from males on treatment with MPA. However, evidence of malformations in offsprings of male kidney transplanted patients (KT) exposed to MPA is limited. Thus, the present study aimed to evaluate the incidence of offspring malformations in children of renal transplanted males under MPA.

**Materials and methods:**

We conducted a retrospective study in which we evaluated the incidence of malformations in descendants from male recipients that were exposed or not to MPA before and at the time of conception. Two groups of patients were evaluated. Those exposed to MPA (MPA group, n = 20) and the non-MPA group (n = 13) that included patients that did not receive AZA (n = 5) and eight that did receive AZA (n = 8) at the time of conception.

**Results:**

A total of forty-nine post-transplant conceptions were identified from 33 different renal transplanted males. MPA was used as the immunosuppressant in 28 of the conceptions. Males from the non-MPA group fathered the other 21 children. Median time from grafting to conception was 6.1 (IQR 2.4–11.1) years, and it was similar between groups. There were eight miscarriage episodes, 2 in the non-MPA group and 6 in the MPA group although differences were not reached. After that, all patients had children without problems. No malformations were detected in any of the 49 regardless whether they were exposed or not to MPA.

**Conclusions:**

No evidence of MPA-associated malformations was observed in descendants of kidney transplanted males on treatment with MPA. Further research is warranted to confirm our findings to properly advice transplanted males keen to procreate.

## Introduction

Kidney transplantation (KT) is the best treatment for end-stage renal disease (ESRD). The development of Immunosuppressive medications has allowed a better graft survival by decreasing graft rejection and preserving the kidney function [1]. Together with tacrolimus, mycophenolic acid (MPA), has become the cornerstone of immunosuppressive regimens in patients with KT. MPA inhibits de novo purine synthesis by blocking the inosine monophosphate dehydrogenase which is directly associated with DNA synthesis [2,3]. As a result, MA prevents the proliferation of T and B lymphocytes [3]. The main side effects of MPA are associated with hematopoietic disturbances [3,4]. However, right after the introduction of MPA, there were reports of fetal malformations in pregnant women on treatment with MPA [3,5,6]. Since MPA is widely used in male and female patients with autoimmune diseases and transplanted recipients, concerns about the safety of its use by women of childbearing age and even men keen on procreating have emerged [7]. Unlike in females, there is limited information about teratogenic effects of MPA in the descendants of males that have procreated while on treatment with MPA after KT. A previous report found similar outcomes of pregnancies fathered by transplant recipients treated with MPA compared to the general population [8]. However, in spite of the more than 20 years of experience using MPA, in 2015, the European Medicines Agency (EMEA) [9] and the Spanish Agency of Medicine and Sanitary Products (AEMPS) [10] warned about the potential teratogenic effects that MPA could induce in the descendants of males receiving MPA, so that males should also use reliable contraceptives methods.

Therefore, the present study aimed to evaluate the incidence of offspring malformations fathered by KT recipients.

## Materials and methods

**Fig 1:**
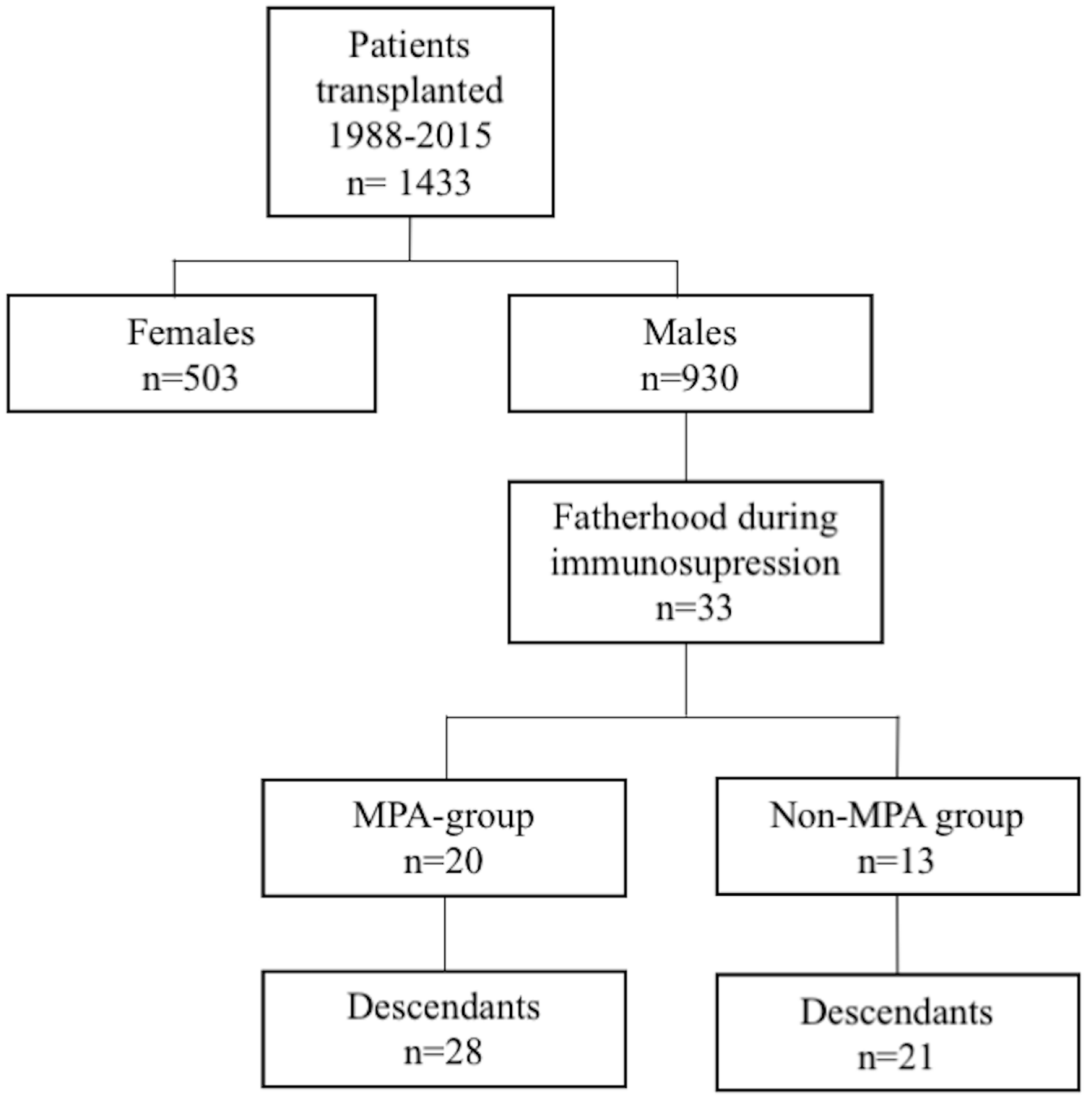
Flow-chart of patients included in the study.

We conducted a longitudinal and retrospective study to evaluate the offspring features of descendants from male kidney transplants recipients. The patients included had received a kidney transplant between August 1988 and March 2015 at Reina Sofia University Hospital in Córdoba (Spain). Fig 1 shows the flow-chart of the patients included.

We collected clinical and demographic variables from male recipients, including those variables related to fertility, such as age at conception, time from kidney transplantation to conception, modality of renal replacement therapy before transplantation, immunosuppressive treatment, body mass index (BMI), and graft function at the time of conception. Information on offspring was collected through a telephone survey that included the following data: number of conceptions after Kidney transplantation, number of term pregnancies, date of birth of children, gender and malformations detected (S1 Dataset and S1 Appendix). Patients were categorized into two groups. Those exposed to MPA (MPA group) and those that did not receive MPA (non-MPA group). The latter group was made up of 5 patients not receiving antimetabolites and eight under Azathioprine (AZA). In all cases, MPA was accompanied by low dose prednisone and either tacrolimus or cyclosporine. All records/information was anonymized and de-identified before analysis. None of the transplant donors were from a vulnerable population, and all donors or next of kin provided written informed consent that was freely given. The study was approved by the local institutional ethics committee (Comité de Ética de la Investigación de Córdoba; ref.3920; Acta 277).

### Statistical analysis

A descriptive statistic of the variables collected was performed. Data of continuous variables were expressed as the mean ± standard deviation or median and interquartile range according to whether they followed or not a normal distribution. Categorical variables are shown as absolute values and percentage. Parametric and nonparametric comparisons of group differences, such as the T-student test or the Mann-Whitney tests were used for quantitative variables where appropriate. Chi-square analysis was used to evaluate differences between proportions. Data were analyzed using SPSS statistics software version 15.0 (SPSS, Inc., Chicago, IL). Two-sided *P* values of <0.05 were considered statistically significant.

## Results

Overall, 1433 patients were transplanted during the study period. A total of thirty-three males fathered 49 children during immunosuppressive consumption (Fig 1). At conception, 20 renal transplanted males were under MPA (MPA group) [64.2%, n = 18 were receiving mycophenolate mofetil while 35.7% (n = 10) were under mycophenolate sodium]. Most of the patients received an isolated kidney allograft (24 in the MPA group and 21 in the non-MPA group), and only four patients with CKD secondary to Type 1 diabetes mellitus received a simultaneous Pancreas-Kidney transplant (SPK) (8.2%).

Table 1 shows the demographic and clinical characteristics of patients evaluated, including variables related to fertility. Whereas the mean age of the patients in the MPA group at the time of conception was 36.9 ± 4.6 years, in the non-MPA group was 34.6 ± 5.7 years (P = 0.16). The main etiology of chronic kidney disease (CKD) was glomerular disease followed by that of CKD unknown etiology. The MPA average mean dose was 1.21 ± 0.3 g/day.

**Table 1:** Demographic and clinical characteristics of transplant recipients.

Recipients	MPA n = 20	Non-MPA n = 13	P*
**Age at Conception (years)** ^#^	36.9 ± 4.6	34.6 ± 5.7	0.16
**Hypertension (n, %)**	14 (66.3)	8 (36.4)	0.61
**Diabetes Mellitus (n, %)**	2 (50%)	2 (50)	0.64
**CKD Etiology** ^a^			
***Unknown*** *(n*, *%)*	4 (9.1)	12 (75.0)	<0.01
*Obstructive Nephropathy*	5 (62.5	3 (37.5)	0.73
*Glomerular disease*	14 (70.0)	6 (30.0)	0.13
*Type 1 Mellitus Diabetes*	4 (100)	0	0.12
**Renal Replacement Therapy**			
*Preemptive kidney transplantation*	1 (3.6)	0	0.38
*Hemodialysis*	21 (75.0%)	21 (18.0)	0.01
*Peritoneal Dialysis*	6 (21.4%)	0	0.02
**BMI (kg/m^2^)** ^b^^,^ ^#^	27.4 ± 4.4	27.0 ± 5.5	0.82
**Median time from transplant to conception (years)** ^#^	5.7 (2.4–8.8)	6.2 (3.1–12.6)	0.31
**Age of mothers (years)** ^#^	31.1 ± 4.9	31.8 ± 3.9	0.85
**Child Weight (g)** ^#^	3298.9 ± 646.4	3148.3 ± 405.8	0.68
**Kidney graft function**			
*Serum Creatinine (mg/dl)*	1.4 ± 0.4	1.8 ± 0.4	<0.001
*Serum Urea (mg/dl)*	49.8 ± 17.7	75.4 ± 18.7	<0.001

^a^ CKD, Chronic kidney Disease;

^b^ BMI, Body Mass Index.

^#^ Mean ±SD, standard deviation.

^¶^ Median and interquartile range.

*The chi-square test or the Mann-Whitney test were used as appropriate.

Regarding the number of conceptions in each male recipient, we found that 13 of the patients fathered only one child, 6 of the patients fathered two children each (one of them twins), and one patient had conceived three children after receiving kidney transplantation in the MPA group. The proportion of girls, 46% (n = 13), and boys, 54% (n = 15) were comparable within this group. In the non-MPA group, five recipients fathered one child, and eight fathered two children each, of whom 5 (23.5%) were girls whereas the remaining 16 (76.1%) were boys.

With regard to comorbidities, hypertension and diabetes proportion were similar in both groups. However, the cause of CKD was unknown in a higher percentage of patients in the non-MPA group compared to the MPA group (Table 1). BMI (P = 0.82), and the age of mothers to these children were also similar between groups (P = 0.85) (Table 1).

The mean period elapsed from transplantation to conceptions was seven years, and it was comparable between groups (7.9 ± 5.3 for the non-MPA group in comparison to 6.3 ± 4.5 for the MPA group; P = 0.31). However, serum creatinine and serum urea were better in the MAP group (P<0.001 for both comparisons) (Table 1).

On the whole, eight miscarriages were reported. Although 2 of them were recorded in the non-MAP group and 6 in the MPA group, no differences were observed (P = 0.43). Nevertheless, term pregnancies were accomplished after that in both groups.

The mean age of descendants at the time of the study was 5.16 ± 3.97 years. In the non-MPA group, one of the children was diagnosed with Down Syndrome right after birth. However, none of the 21 children in the non-MPA group as none in the MPA group showed physical malformations. Birth weights of descendants were comparable between groups.

## Discussion

The present study aimed to evaluate whether the exposure to MPA was associated with a higher incidence of malformations in the offsprings of male KT recipients. Our results show that among male recipients on treatment with MA who had descendants after transplantation, the rate of malformations was not increased compared to that of the recipients that were not exposed to MPA at conception.

Current immunosuppressive medications have improved graft survival. Such medications prevent T and B lymphocytes proliferation [2]. The combination of corticosteroids, calcineurin inhibitors such as tacrolimus and MPA are the hallmark of current immunosuppressive regimens. Side effects of immunosuppressive medications resulting from DNA disturbances constitute a significant concern [3,5]. MPA may promote disturbances in DNA synthesis through indirect mechanisms [11,12]. Recent reports have described a higher incidence of malformations in the descendants of females on treatment with MPA [2,3]. However, data regarding such an effect in the descendants of male KT recipients is limited.

Our results show that no malformations were recorded in the descendants of male KT recipients under MPA. Similarly, no evidence of malformations was recorded in the non-MPA group. Therefore, like others [12], we show that antimetabolite exposure in general, and specially MPA might not be associated with a higher proportion of malformations in the descendants fathered during its consumption.

Regarding miscarriage episodes, there were no differences between groups. Although evidence suggests that the risk of abortions may be increased in female recipients under MPA, no solid recommendation exists for males, unlike females. However, the rate of abortions observed in our study was not different from that of the general population worldwide [13]. Furthermore, conceptions and pregnancies were accomplished with no medical problems after that, so that, it was considered that these miscarriage episodes were unrelated to MPA. A recent study is also in line with our results since paternal exposure to MPA was not associated with an increase in the risk of malformations in the descendants of male KT recipients [12].

Because of the transcendental nature of such recommendations for male KT recipients, the EMEA has now revised and updated their warning, so that although the risk of genotoxicity cannot be excluded entirely, no longer simultaneous reliable contraception is mandatory for both recipients and their partners [14]. Data on the better graft function of patients under MPA compared to the non-MPA group are not surprising because of the already proven benefits of MPA compared to AZA regimes after KT [1].

Our study has some limitations. First, this is a retrospective, and observational single-center study and some isolated cases could have been missed. However, because ethical reasons would prevent randomized trials, the observational collection of data is the only source of information that can be provided. Second, we included a small number of conceptions which is explained by the limited percentage of fertile male KT recipients in our center.

In conclusion, MPA was not associated with an increased incidence of malformation in descendants of male KT recipients. Our results improve our scientific acknowledge about the currently limited evidence of the non-teratogenic effects of this kind of medications to properly counseling KT male recipients to be keen on reproducing.

## Supporting information

S1 Appendix Survey questionnaire. (PDF)

S1 Dataset Compilation of data from patients included in the analysis. (CSV)
