# Supplementary material for: Administration of mycophenolic acid is not associated with malformations in descendants from kidney transplanted males
Source: PLoS One. 2018 Sep 12;13(9):e0202589. doi: 10.1371/journal.pone.0202589 (PMC6135357; doi:10.1371/journal.pone.0202589)
Supplement: S1 Appendix — (PDF) [file pone.0202589.s001.pdf]

S1 Appendix.

Survey questionnaire

Tittle of the study: "Risk of malformations induced by Mycophenolic acid on descendants from renal transplanted males."

Dear patient, as a transplant recipient you are followed-up in our facilities by our transplantation team with the aim of evaluating your graft function periodically. As you may know, immunosuppressive medications, beyond preventing the rejection of your graft, they may also have secondary effects. One of the most prescribed medications worldwide after kidney transplantation is the mycophenolic acid. This medication has been associated with an increase in the rate of malformations in descendants when taken by women during pregnancy. However, evidence demonstrating such a fact in children fathered by renal transplanted males is limited.

You should be advised that your participation is voluntary and that your answers would be completely anonymous. This questionnaire does not include information that could be associated with you. All data are anonymized and de-identified before analysis.

Please, answer the question as bright as possible.

Questions:

1. **Do you have children?**
2. **If the former answer is affirmative, then how many did you have?**
3. **At the time of conception were you an active smoker? Were you receiving dialysis and if so, was it hemodialysis or peritoneal dialysis? Had you received transplantation before?**
4. **Have you ever visited a specialist in reproductive medicine?**
5. **Is your answer to the prior question is affirmative, was any there any fertility problem identified?**
6. **If your answer to the former question is affirmative, then did you received any specific treatment or a diagnostic procurement?**

7. **With regard to the mother of your children, could you please tell us the following information:**
- Date of birth
- Weight and size
- Was your partner active smoker at conception?
- Is there any disease to report?
- Any specific treatment consumption before or during pregnancy?
- Did your partner require any consultation to a fertility clinic?
- If your answer to the previous answer is affirmative, then was any problem fertility detected?
- If your answer to the last question is affirmative, then did she require any specific treatment for fertility

8. **Please specify:**
- Date of birth of any of your children
- Were they term pregnancies
- Gender of each of the descendants
- Birth weight of each of them
- Was any illness or malformations identified during pregnancy?
- Was any disease of malformation identified at birth or right after?

9. **Did your partner suffer any abortion?**

10. **If your answer to the previous question is affirmative then,**
- How many of them?
- Were there spontaneous or medically indicated?
- Was any disease or malformation identified? Please mention which one.
